# Equity in the Allocation of General Practitioner Resources in Mainland China from 2012 to 2019

**DOI:** 10.3390/healthcare11030398

**Published:** 2023-01-31

**Authors:** Yingjie Fu, Jian Wang, Jiyao Sun, Shuo Zhang, Derong Huang

**Affiliations:** 1 Center for Health Management and Policy Research, Cheeloo College of Medicine, School of Public Health, Shandong University, Jinan 250012, China; 2 NHC Key Lab of Health Economics and Policy Research, Shandong University, Jinan 250012, China; 3 Social Statistics, Manchester Institute for Collaborative Research on Ageing (MICRA), The University of Manchester, HBS Building, Manchester M13 9PL, UK; 4 Cathie Marsh Institute for Social Research (CMI), The University of Manchester, HBS Building, Manchester M13 9PL, UK

**Keywords:** general practitioners, resource allocation, equity, mainland China

## Abstract

Background: General practitioners (GPs) play a vital role in primary health care services and promoting the health equity of residents, but there is a paucity of evidence on equity in the allocation of GP resources in mainland China. This study explores equity in the allocation of GP resources from 2012 to 2019 in mainland China. Methods: We used GP data from 31 provinces, autonomous regions, and municipalities in mainland China. Lorenz curves, Gini coefficients, Theil indices, and agglomeration degree were used to analyze the data. Results: The total number of GPs in China was 365,082 in 2019, which corresponded to 2.61 GPs per 10,000 residents and accounted for 9.44% of the total number of practicing doctors in 2019. From 2012 to 2019, the Gini coefficient of GP allocation based on population decreased from 0.3123 to 0.1872. However, the Gini coefficient based on geographical area was maintained at 0.7108–0.7424. The Theil index of GP allocation based on population decreased from 0.0742 to 0.0270, but GP allocation based on geographical area was maintained at 0.5765–0.6898. The intra-regional contribution rates were higher than the inter-regional rates. The agglomeration degree based on geographical area and population decreased in the eastern region but increased in the central and western regions. Conclusions: The number of Chinese GPs has increased rapidly in recent years, but the distribution of GPs across China is uneven. In the western and middle regions, there is a relative shortage. Equity in the allocation of GP resources based on population was far greater than that based on geographical area. In the future, the tough issue of inequitable GP resource allocation should be resolved by comprehensive measures from a multidisciplinary perspective.

## 1. Introduction

The World Health Organization refers to health as a basic right of human beings and one of the most precious treasures in life [1]. In other words, everyone has the right to basic medical and health services. Equity of health services is a core aim of primary health care. Equity in health resource allocation (HRA) is a basic condition of health equity and plays an important role in providing every individual with access to primary health care services [2]. General practitioners (GPs) have comprehensive medical knowledge and skills. They provide integrated services, including prevention and essential health care; diagnosis, treatment, and referral of common diseases; rehabilitation and management of chronic diseases; and health management [3]. GPs can treat 80% to 90% of common diseases, frequently occurring diseases, senile diseases, and chronic diseases in primary medical institutions. They are known as the gatekeepers of residents’ health [4]. After the outbreak of the COVID-19 pandemic, primary-level medical institutions and GPs, as the first line of defense, played a fundamental and indispensable role in pandemic prevention and control [5].

A new round of health care reforms to improve equity in HRA was launched by the Chinese government in 2009. Its aim was to maintain basic-level health care, whilst reinforcing grassroots-level health care, with an emphasis on strengthening teams of personnel working in grassroots health care, especially GPs [3]. A national policy document concerning the establishment of a GP system published in 2011 stated that China would gradually standardize GP training to a “5 + 3” model [6]. This system requires a prospective GP to attend 5 years of undergraduate education in clinical medicine (including traditional Chinese medicine) and then complete 3 years of standardized GP training. GP training and general practice in China are still in their infancy, and there is a severe shortage [7]. An important aspect of the health system reforms is to establish a network of GPs, with GPs at the center of primary health care teams. Achieving this aim can improve the level of primary health care services and access to doctors, as well as reduce medical costs [7]. To attract individuals to become GPs and, thus, improve the quantity and quality of GPs, the government also issued a series of policy documents and measures related to GP development, training, and incentive mechanisms.

Several methods have been employed to evaluate equity in the allocation of GP resources. For example, the Robin Hood index was used to analyze equity in the allocation of GP resources in Australia [8]. A principal component analysis, the general index of deprivation, and equity-adjusted share were used to evaluate equity in resource allocation for health in Ghana [9]. Lorenz curves, the Gini coefficient, the Atkinson index, the Robin Hood index, and decile ratios were used to analyze equity in the allocation of GP resources in Albania [10].

In China, previous studies have mainly focused on analyzing equity in the allocation of GP resources at the national level or in a particular region at a specific time. Furthermore, most studies have used Lorenz curves, Gini coefficients, Theil indices, and concentration indices to analyze equity in the allocation of GPs in China as a whole or in a particular area. For example, the health resource density index, the Gini coefficient, and the Theil index were used to conduct an analysis on the allocation of Chinese GPs in 2012 and 2014 [11]. Aggregation degree was used to evaluate the allocation of GPs in 2015 [12], and both the Gini coefficient and aggregation degree were used to evaluate equity in the allocation of GPs from 2012 to 2017 [13]. A concentration index and the Theil index were used to evaluate changes in the equity of GP resources from 2013 to 2017 [14], and a concentration index and the Gini coefficient were used to analyze the allocation of GPs in Guangxi [15]. In addition, the Gini coefficient and Lorenz curves were used to analyze equity in the allocation of GP resources in Shandong from 2013 to 2018 [16]. Each method has its own advantages, but these methods do not take into account the impact of geographic and demographic factors on HRA [17,18]. Lorenz curves reflect equity in HRA when combined with Gini coefficients, but this analysis can only determine the overall degree of difference [17]. The Theil index incorporates the contribution rates within and between groups when measuring the main factors causing disparities [17]. Concentration indices can be used to measure overall inequity but do not include resource delivery variables [17]. In this study, agglomeration degree is used. This method considers equity in HRA based on population distribution and geographic distribution and also analyzes regional equity differences [18].

The current study is a comprehensive analysis of HRA equity at the national and regional level. We use Lorenz curves, Gini coefficients, Theil indices, and an agglomeration analysis to evaluate equity in the allocation of GP resources from 2012 to 2019 in mainland China using the latest nationwide data. The results of the study can be used to inform public health policy and optimize GP resource allocation.

## 2. Methods

### 2.1. Data Sources

This study used GP data from 31 provinces, autonomous regions, and municipalities in mainland China. We obtained the year-end population and jurisdiction area of each region from the China Statistical Yearbook (2013–2020) [19,20,21,22,23,24,25,26]. GP data were obtained from the China Health and Family Planning Statistical Yearbook (2013–2020) [27,28,29,30,31,32,33,34]. The number of GPs referred to the total number of practitioners who were either registered as GPs or obtained a GP training certificate. We distinguished eastern, central, and western regions according to the China Health and Family Planning Statistical Yearbook 2020. The eastern region included 11 provinces and municipalities (Beijing, Tianjin, Hebei, Liaoning, Shanghai, Jiangsu, Zhejiang, Fujian, Shandong, Guangdong, and Hainan). The central region included eight provinces (Shanxi, Jilin, Heilongjiang, Anhui, Jiangxi, Henan, Hubei, and Hunan). The western region included 12 provinces, autonomous regions, and municipalities (Inner Mongolia, Chongqing, Guangxi, Sichuan, Guizhou, Yunnan, Tibet, Shaanxi, Gansu, Qinghai, Ningxia, and Xinjiang).

### 2.2. Data Analysis

#### 2.2.1. Lorenz Curves and Gini Coefficients

A Lorenz curve is a graphical representation of income inequity or wealth inequity developed by the American economist Max Lorenz in 1905 [17]. The more curved the Lorenz curve, the more unequal the distribution. We ranked 22 provinces, as well as 5 autonomous regions and 4 municipalities under their jurisdiction, according to the number of GPs per capita. Lorenz curves were then created according to the distribution of the service population by taking the cumulative percentage of GPs as the vertical coordinate and the cumulative percentage of the population as the horizontal coordinate. Moreover, the 22 provinces, 5 autonomous region, and 4 municipalities were ranked according to the number of GPs per square kilometer. Lorenz curves distributed by geographical area were created by taking the cumulative percentage of GPs as the vertical coordinate and the cumulative percentage of the population as the horizontal coordinate. Calculated from a Lorenz curve, a Gini coefficient evaluates the equity of income distribution as defined by the American economist Albert Hirschman [10]. A Gini coefficient, which has a value between 0 and 1, is an important parameter used to represent income distribution differences among individuals on a global scale. It has also been widely used to evaluate the relationship between inequality and health [17]. A Gini coefficient of less than 0.2 means absolute equality. A value of 0.2–0.3 means relative equality, while 0.3–0.4 means adequate equality, 0.4–0.5 means relative inequality, and more than 0.5 means severe inequality [17].

#### 2.2.2. Theil Index

The Theil index was developed by the economist Henri Theil in 1967, who used entropy theory to evaluate the equity of income [35]. The Theil index ranges from 0 to 1. The smaller the value, the more equitable the different regions. The Theil index was originally used to measure income equity but is increasingly used to measure HRA equity. The Theil index equation is as follows:

$$T=\sum_{i=1}^{n}P_{i}\log\frac{P_{i}}{Y_{i}}.$$

where *P_i_* is the proportion of the population in a region relative to the total population, and *Y_i_* is the total number of health resources in a region. The total Theil index can be divided into two groups called the “within group” and the “between groups.” The decomposition formula of the Theil index is as follows:

$$T_{\mathrm{intra}}=\sum_{g=1}^{k}P_{g}T_{g},T_{\mathrm{inter}}=\sum_{g=1}^{k}P_{g}\log\frac{p_{g}}{Y_{g}}$$

*T* = *T*_intra_ + *T*_inter_*. T*_intra_ represents the degree of HRA equity within an area, and *T*_inter_ represents the degree of HRA equity between different areas. *P_g_* and *Y_g_* have the same meanings as *P_i_* and *Y_i_*, respectively. The contributions of the “within group” and “between groups” can be calculated by dividing *T* [17].

### 2.3. Agglomeration Analysis

We used an agglomeration analysis to measure the degree of health resources in a particular region and the differences between groups. The agglomeration analysis of GP resources was carried out in two dimensions based on geographical area and population. The equation for agglomeration degree based on geographical area was HRAD_i_ = (HR_i_/A_i_)/(HR_n_/A_n_), where HR_i_ represents the number of GPs in region i; HR_n_ represents the total number of GPs in China; A_i_ represents the land area in region i; and A_n_ represents the land area in China. The equation of agglomeration degree based on population was HRAD_i_/PAD_i_ = (HR_i_/P_i_)/(HR_n_/P_n_), where PAD_i_ represents the population agglomeration degree in region i; HR_i_ and HR_n_ have the same meanings as above; P_i_ represents the population number in region i; and P_n_ represents the total population number for China [18].

The agglomeration analysis was evaluated using the following criteria. If the agglomeration degree based on the geographical area was 1, the allocation of GPs was absolutely equitable in this region. If the agglomeration degree based on the geographical area was close to 1, the equity of distribution in terms of the geographical area was adequate. Similarly, if the agglomeration degree based on population size was 1, the allocation of GPs was absolutely equitable in this region. If the agglomeration degree based on population size was close to 1, the equity of distribution in terms of population was adequate. It should be noted that, if the agglomeration degree based on geographical area or population size was slightly greater than 1, it indicated relatively equitable GP allocation. If the agglomeration degree was far greater than 1, it indicated a greatly excessive concentration of GP allocation. If the agglomeration degree was less than 1, it indicated high inequity in GP allocation or that GP allocation was insufficient [18].

## 3. Results

We analyzed the distribution trends and equity of GP resources in mainland China from 2012 to 2019 at national and regional levels using multiple parameters, including Gini coefficients, Lorenz curves, Theil indices, and agglomeration degrees.

### 3.1. The Distribution Trend of GPs

The total number of GPs in China increased from 109,794 in 2012 to 365,082 in 2019, which was an increase of 232.52% and an average annual growth rate (AAGR) of 18.73%. From 2012 to 2019, the AAGR of GPs per 10,000 inhabitants in China was 18.19%, and the AAGRs in the eastern, central, and western regions were 15.59%, 22.64%, and 19.77%, respectively. For specific areas, Tibet, Jilin, and Guizhou had the highest AAGRs of 49.43%, 29.84%, and 28.96, respectively.

Table 1 shows that, in 2019, there were 365,082 GPs in China, with 192,116 in the eastern region, 94,847 in the central region, and 78,117 in the western region, accounting for 52.62%, 25.98%, and 21.40%, respectively. The average number of GPs per 10,000 population in China was 2.61 (Table 2). From the perspective of different regions, the average numbers of GPs per 10,000 population were 3.28 in the eastern region, 2.17 in the central region, and 2.05 in the western region. For specific provinces, autonomous regions, and municipalities in mainland China, the numbers of GPs per 10,000 population in Jiangsu, Zhejiang, Shanghai, and Beijing exceeded 4, with Jiangsu showing the highest value of 5.90. In addition, except for Tianjin, Jilin, and Guangdong, other provinces, autonomous regions, and municipalities had values below the national average.

According to the analysis of GPs in mainland China, GPs as a proportion of all practicing doctors were 4.20% in 2012, increasing to 9.44% in 2019. The proportion of GPs in the western region was the lowest, at only 7.90% in 2019, and the highest in the eastern region (10.86%; Table 3). Table 4 shows that the nationwide registration rate of GPs increased from 33.86% in 2012 to 57.69% in 2019. The registration rate grew most rapidly in the eastern region (36.19% to 64.37%). It also increased from 2012 to 2019 in the western region (26.39% to 48.44%) and central region (33.99% to 51.80%). From the perspective of institutional distribution, most of the registered GPs were in community and township hospitals, while the majority of those who obtained GP training certificates were in township hospitals (Table 5).

### 3.2. Lorenz Curves and Gini Coefficients

**Figure 1:**
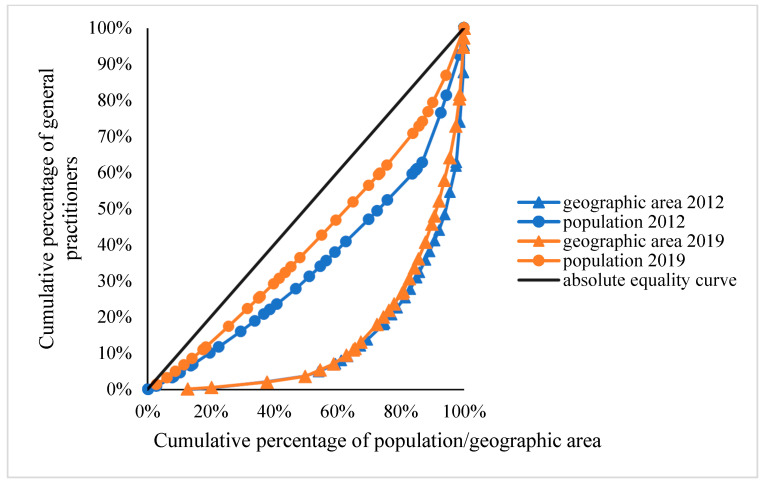
The Lorenz curves of GPs in 2012 and 2019.

Figure 1 illustrates the Lorenz curves based on population and geographical area. The Lorenz curves of GP allocation based on population were close to the absolute equity curve, while the Lorenz curves based on geographical area deviated considerably from the absolute equality curve. Table 6 shows that the Gini coefficient of GP allocation based on population decreased from 0.3123 in 2012 to 0.1872 in 2019. However, the Gini coefficient of GP allocation based on geographical area remained stable at 0.7108–0.7424. These findings demonstrate that GP allocation in mainland China based on population had relative equity, and even absolute equity in 2019, but that GP allocation based on geographical area had severe inequality.

### 3.3. The Theil index

Table 7 shows that the Theil index of GP allocation based on population decreased from 0.0742 to 0.0270 between 2012 and 2019, but when based on geographical area, it was maintained at 0.5765–0.6898. The Theil index based on geographical area showed a slight upward trend between 2015 and 2018. Moreover, the Theil index showed a consistent trend with the Gini coefficient, indicating that the equity findings were similar using both approaches. Worse equity in the allocation of GPs based on population and geographical area was derived from intra-regional differences. The intra-regional contribution rate based on population was approximately equal to 60%, and that based on geographical area was 55%. Subsequently, we used decomposition of the total Theil index to evaluate intra-regional differences (Table 8 and Table 9). From the perspective of population dimension, differences in the intra-central and western regions decreased, whereas those in the intra-eastern region increased. The Theil index was the largest in the eastern region based on population dimension. However, internal differences in the western region contributed the most to the geographical area dimension, which was approximately 96%. The Theil index of GPs was largest in the western region and smallest in the central region. The findings indicate that the worse equity in the allocation of GPs based on population was derived from the eastern region, and the worse equity based on geographical area was in the western region.

### 3.4. Agglomeration Analysis

**Figure 2:**
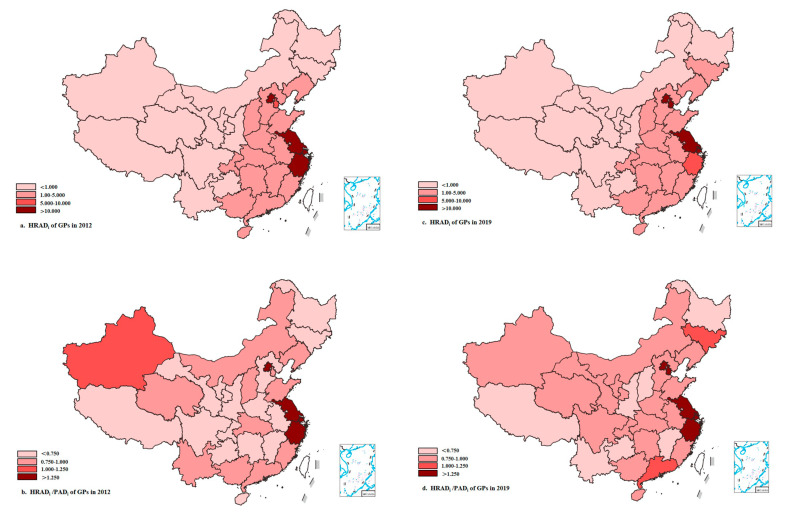
The HRAD_i_ and HRAD_i_/PAD_i_ of GPs in 2012 and 2019.

The agglomeration degrees based on geographical area allocation are shown in Table 10. From 2012 to 2019, the agglomeration degree decreased from 5.316 to 4.626 in the eastern region, which was far greater than 1, indicating an excessive concentration of GPs. Moreover, it increased from 1.150 to 1.478 in the central region, which was greater than 1, indicating relatively equitable GP allocation. Although the agglomeration degree increased from 0.272 to 0.301 in the western region, the value was much less than 1, suggesting inequitable GP allocation. From the perspective of different provinces, autonomous regions, and municipalities, the agglomeration degree was relatively high in Shanghai and Beijing but declined in 2012 to 2019. In addition, the values exceeded 10 in Jiangsu and Zhejiang, indicating that GP allocation was over-concentrated based on the geographical area. The agglomeration degrees in Tibet, Qinghai, Xinjiang, Inner Mongolia, Gansu, Heilongjiang, Ningxia, Guizhou, Jilin, Yunnan, Shaanxi, and Sichuan were relatively low, with values less than 1, indicating high inequity of GP allocation based on geographical area. To identify geographical differences in the allocation of GP resources more clearly, a distribution map of the agglomeration level was created. Figure 2 shows that the agglomeration degree in the eastern region was much higher than those in the central and western regions.

The agglomeration degrees based on population allocation are shown in Table 11. In 2019, the agglomeration degrees in the eastern, central, and western regions were 1.262, 0.835, and 0.787, respectively. The agglomeration degrees in the central and western regions increased from 2012 to 2019. This finding shows that GP resource allocation based on population was insufficient in the central and western regions. Although the agglomeration degree in the eastern region decreased from 2012 (1.460) to 2019 (1.262), the values were greater than 1, which indicated that GPs were too concentrated based on the population. From the analysis of different provinces, autonomous regions, and municipalities, the resource allocation of GPs in Chongqing (0.999) had absolute equity. The agglomeration degrees in Tianjin (1.125), Jilin (1.077), Guangdong (1.066), and Qinghai (0.958) approached 1, indicating that their GP resource allocation had equity based on the population. Moreover, the agglomeration degrees in Jiangsu (2.268), Zhejiang (1.801), Beijing (1.654), and Shanghai (1.572) were the highest values and were far greater than 1, indicating that the GPs in these areas were too concentrated. However, the agglomeration degrees of 24 provinces and autonomous regions were less than 1, indicating that GP resources were relatively scarce, and the population allocation was insufficient. In addition, we found that GP resources in China were gradually becoming more equitable based on the population.

## 4. Discussion

After long-term development, China has established a relatively mature medical and health service system including primary health services. From a nationwide perspective, this study comprehensively evaluated trends in GP resource allocation in mainland China from 2012 to 2019.

The number of GPs has rapidly increased in China, but regional differences are large, and the training system still needs to be improved. According to a government report concerning GP training and the use of GPs issued in 2018, there should be 2–3 qualified GPs for every 10,000 urban and rural residents by 2020, as well as five qualified GPs for every 10,000 urban and rural residents by 2030 [36]. By the end of 2019, the number of GPs per 10,000 population reached 2.61. Although the relevant policy goal for 2020 was achieved, this standard is far from the international standard, which states that each GP should be responsible for 2000 residents. In addition, the current total allocation is still insufficient. This study showed that most of the GPs in China were doctors who obtained GP qualification certificates after job-transfer training and that this situation occurred mainly in community and township hospitals.

Job-transfer training to create GPs is not conducive to the effective promotion of primary diagnosis by family doctors and hierarchical diagnosis and treatment. After continuous exploration and practice in recent years, GP team construction has progressed in China. However, the growing demand for basic medical care means that the current quantity and quality of GPs and the training system still needs to be improved. To meet these objectives, the government should continue to implement existing policies and improve the training system for GPs. In terms of training, fragmented training should be avoided, and the training of GPs should be gradually unified into the “5 + 3” standardized training model. Furthermore, an “Internet+” approach should be used to build a GP training information platform and develop general practice [37,38]. For example, the “MOOC (Massive Open Online Courses)-flipped classroom,” which is a hybrid teaching model combining both online and offline training, could solve problems related to the high cost and uneven quality of traditional GP training.

The allocation of GPs is unbalanced and large regional differences exist in China. In our nationwide study, the total number of GPs, the number of GPs per 10,000 population, and the agglomeration degree of GPs in eastern China were higher than those in central and western China, and regional differences were large. These findings are consistent with those of Zhou et al. [39], Liu and Yin [11], Zhang et al. [40], and Liu et al. [41], who have highlighted advantages in the eastern region, with large regional differences in health resources. The current situation is not conducive to the sustainable development of GP systems and general practice in China. Promoting the establishment of general practices in the central, western, and rural areas is urgent. First, the government should improve the working conditions of GPs in grassroots areas and increase the attractiveness of becoming a GP. Second, they should improve the incentive mechanisms of the GP system and appropriately tilt them toward the central and western regions and to rural areas. Third, information construction should be accelerated, actively promoting an “Internet + GP” model to improve the interconnectivity of high-quality medical resources [42]. Lastly, interaction with residents through the Internet can help to create good doctor–patient relationships, improve the social status of GPs, and enable GPs to truly become the gatekeepers of resident health.

This study revealed big differences in equity in the allocation of GPs in different regions of China. The Gini coefficient of GP allocation in mainland China based on population showed relatively equity, and even absolutely equity in 2019, but the coefficient based on geographical area demonstrated severe inequality. The results indicated that equity in the allocation of GP resources based on population distribution was better than that based on geographical area. The Theil indices of GPs showed the same trend as that of the Gini coefficient. Contribution rates after the decomposition of the total Theil index can help us better understand the reasons for the inequity of HRA. The results showed that worse equity in the allocation of GPs based on population and geographical area were derived from intra-regional differences. Specifically, the inequality in the allocation of GPs based on population was derived from the eastern region and that based on geographical area stemmed from the western region. However, HRA in China at a nationwide level, without considering intra-regional differences, showed a trend toward more equitable development in recent years [11].

The findings of the agglomeration analysis reflected differences between different regions. GP resource allocation in the central and western regions was insufficient, while in the eastern region, the resource allocation was too concentrated. By taking the agglomeration analysis in 2019 as an example, the agglomeration degrees based on population and geographical area allocation were far greater than 1 in Shanghai, Beijing, Jiangsu, and Zhejiang, suggesting that the allocation of GPs was excessively concentrated. The agglomeration degrees based on geographical area were less than 0.4 in Tibet, Qinghai, Xinjiang, Inner Mongolia, Gansu, and Heilongjiang. Moreover, the agglomeration degrees based on population were less than 1 in 24 provinces and autonomous regions.

The equity of areas such as Tibet, Qinghai, Xinjiang, Inner Mongolia, Gansu, and Heilongjiang was insufficient based on geographical or population allocation, and GPs were scarce. There may be several reasons for this situation. One reason is the poor economic conditions in these areas and the other is that the western region is an unattractive place to live because of the thin air, low pressure, and low oxygen content. Additionally, existing policy documents for health resource planning are still based on population and administrative division to allocate health resources. There are a large number of sparsely populated plateaus and mountains in western China, which makes GP resources distributed according to geographical area extremely scarce. Therefore, the Chinese government could strengthen macro-control and guidance to encourage GPs to move between different regions or from urban to rural areas, thereby increasing the accessibility of basic health services for residents in various regions. Furthermore, the government could optimize HRA by considering the impacts of population size, geographical area, economic development level, service demand, service radius, and capacity on the accessibility of health resources and formulating policies according to local conditions [43,44]. For instance, in the eastern region and some central plain areas, the equity of health resources based on population distribution should be considered. However, in the sparsely populated western and central regions, more attention should be paid to the equity of health resources based on geographical distribution, and the uptake rate of health resources should be improved [18].

In this study, equity was evaluated using the latest available nationwide data; furthermore, agglomeration degree was combined with data mapping to visualize differences in equity. However, some limitations existed. First, this study evaluated equity in the allocation of GP resources based on population and geographical area without considering the interactions among population size, geographical area, and economic development level. Second, this study evaluated equity in the allocation of GP resources based on the hypothesis of resource homogeneity without considering differences in service quality and service capacity for different GPs.

## 5. Conclusions

This study provided empirical research on equity in the allocation of GP resources in mainland China based on authoritative data. The results showed that the number of Chinese GPs increased rapidly in recent years, but the total allocation was still insufficient. Equity in the allocation of GP resources based on population distribution improved year by year. However, equity distribution based on geographical area was inadequate. Moreover, the distribution of GPs in different regions was uneven, with large regional differences. In the eastern region, there was an over-concentration of GP resources, while in the western and middle regions, there were relative shortages of GPs based on both population and geographical area. In the future, focus on the training and assessment mechanisms of GPs is needed in order to achieve simultaneous improvement in the quantity and quality of GPs. In addition, the Internet should be used to full effect by actively promoting “Internet + GP,” improving the social status of GPs, and making GPs true gatekeepers of resident health. Finally, the government should continue to strengthen macro-control and guidance of the allocation of GP resources.

## Figures and Tables

**Table 1:** Number of GPs in 2012–2019 in China.

Year	Total	East	Middle	West
2012	109,794	66,401	22,192	21,201
2013	145,511	84,464	29,674	31,373
2014	172,597	96,979	39,020	36,598
2015	188,649	104,015	45,344	39,290
2016	209,083	116,537	49,944	42,602
2017	252,717	139,473	63,269	49,975
2018	308,740	170,362	75,302	63,076
2019	365,082	192,116	94,847	78,119

**Table 2:** Number of GPs per 10,000 in 2012–2019 in China.

Area	2012	2013	2014	2015	2016	2017	2018	2019	AAGR (%)
Total	0.81	1.07	1.27	1.37	1.51	1.82	2.22	2.61	18.19
Eastern region	1.19	1.50	1.71	1.83	2.03	2.42	2.93	3.28	15.59
Middle region	0.52	0.70	0.91	1.05	1.16	1.46	1.73	2.17	22.64
Western region	0.58	0.86	0.99	1.06	1.14	1.33	1.66	2.05	19.77
Beijing	3.93	4.00	3.82	3.81	3.87	3.96	4.11	4.30	1.29
Tianjin	0.77	0.97	1.07	1.39	1.54	2.41	2.65	2.92	20.98
Hebei	0.48	0.92	1.17	1.25	1.25	1.33	1.49	2.42	26.00
Shanxi	0.71	0.81	0.99	1.10	1.13	1.72	1.60	1.75	13.75
Inner Mongolia	0.67	0.95	1.17	1.23	1.26	1.58	1.93	2.28	19.12
Liaoning	0.75	0.80	0.86	0.83	0.96	1.44	2.07	2.49	18.70
Jilin	0.45	0.61	0.84	1.05	1.24	1.89	1.84	2.80	29.84
Heilongjiang	0.54	0.75	0.97	1.13	1.17	1.19	1.49	1.76	18.39
Shanghai	2.24	2.47	2.85	3.04	3.29	3.51	3.56	4.09	8.98
Jiangsu	1.90	2.22	2.48	2.61	3.15	3.43	5.94	5.90	17.57
Zhejiang	2.24	3.10	3.57	3.90	4.04	5.39	4.54	4.68	11.10
Anhui	0.53	0.72	1.12	1.20	1.39	1.67	2.04	2.37	23.86
Fujian	0.69	0.96	1.13	1.33	1.49	1.76	2.08	2.30	18.77
Jiangxi	0.46	0.54	0.66	0.73	0.79	1.14	1.21	1.44	17.71
Shandong	0.70	0.79	0.92	1.01	1.14	1.36	1.73	2.09	16.91
Henan	0.50	0.68	0.89	1.09	1.27	1.63	2.13	2.36	24.82
Hubei	0.65	0.87	1.05	1.19	1.19	1.52	1.84	2.17	18.79
Hunan	0.39	0.59	0.75	0.90	0.96	1.03	1.28	2.42	29.79
Guangdong	0.75	1.11	1.34	1.38	1.67	2.03	2.44	2.77	20.52
Guangxi	0.66	0.86	0.95	0.97	1.05	1.28	1.62	2.15	18.38
Hainan	0.47	0.65	0.81	0.96	1.08	1.22	1.45	2.07	23.59
Chongqing	0.55	0.74	0.84	0.95	1.03	1.26	2.05	2.60	24.85
Sichuan	0.58	1.11	1.21	1.27	1.25	1.37	1.61	2.13	20.42
Guizhou	0.30	0.43	0.69	0.89	1.04	1.40	1.73	1.78	28.96
Yunnan	0.69	0.91	0.87	0.90	0.99	1.09	1.32	1.81	14.77
Tibet	0.11	0.21	0.34	0.50	0.61	0.73	1.02	1.83	49.43
Shanxi	0.49	0.53	0.73	0.56	0.72	0.93	1.29	1.37	15.82
Gansu	0.54	0.82	1.05	1.27	1.45	1.46	1.83	2.26	22.69
Qinghai	0.81	1.31	1.51	1.63	1.67	2.06	2.18	2.49	17.40
Ningxia	0.40	0.60	0.71	0.85	0.97	1.36	1.86	2.16	27.24
Xinjiang	0.86	1.20	1.45	1.57	1.68	1.81	2.05	2.17	14.14

**Table 3:** The proportion of GPs in practice (assistant) as physicians in China in 2012–2019 (%).

Area	2012	2013	2014	2015	2016	2017	2018	2019
Total	4.20	5.21	5.21	6.21	6.55	7.45	8.56	9.44
Eastern region	5.65	6.70	6.70	7.62	8.09	9.09	10.32	10.86
Central region	2.85	3.60	3.60	4.98	5.27	6.34	7.18	8.56
Western region	3.20	4.42	4.42	5.15	5.30	5.83	6.95	7.90

**Table 4:** The registration rate of GPs in China in 2012–2019 (%).

Area	2012	2013	2014	2015	2016	2017	2018	2019
Total	33.86	32.58	37.17	36.24	37.13	38.08	50.79	57.69
Eastern region	36.19	36.13	40.45	39.94	40.79	41.75	57.78	64.37
Central region	33.99	31.11	34.96	34.8	35.57	35.73	44.63	51.8
Western region	26.39	24.39	30.84	28.11	28.93	30.82	39.26	48.44

**Table 5:** Institutional distribution and registration of GPs in China in 2012–2019.

Year	Number of Registrations				Number of Training Certificates			
	Total	Hospital	Community Hospital	Township Hospital	Total	Hospital	Community Hospital	Township Hospital
2012	37,173	5817	18,502	12,304	72,621	15,257	29,361	26,253
2013	47,402	6260	23,488	16,836	98,109	19,498	36,693	29,989
2014	64,156	9395	31,202	22,594	108,441	21,033	37,712	47,702
2015	38,364	8935	33,169	25,434	120,285	22,446	40,119	55,541
2016	77,631	9517	36,513	30,718	131,452	25,137	41,824	62,073
2017	96,235	11,223	41,327	41,181	156,482	38,177	42,606	69,719
2018	156,800	20,966	56,506	64,117	151,940	30,105	39,097	70,421
2019	210,622	26,931	68,001	90,244	154,460	33,568	35,840	71,414

**Table 6:** The Gini coefficients of GP allocation in 2012–2019.

Variable	2012	2013	2014	2015	2016	2017	2018	2019
Population	0.3123	0.2871	0.2565	0.2463	0.2445	0.2423	0.2383	0.1872
Geographical area	0.7424	0.7247	0.7176	0.7152	0.7194	0.7225	0.7259	0.7108

**Table 7:** Theil indices of Chinese GPs in 2012–2019.

Year	Theil Index		Inter-Region Theil Index		Intra-Region Theil Index		Intra-Region Contribution Rate (%)	
	Population	Geographical Area	Population	Geographical Area	Population	Geographical Area	Population	Geographical Area
2012	0.0742	0.6898	0.0324	0.3088	0.0418	0.3810	56.33	55.10
2013	0.0604	0.6406	0.0254	0.2835	0.0350	0.3571	57.95	55.74
2014	0.0486	0.6133	0.0190	0.2750	0.0296	0.3383	60.91	55.16
2015	0.0459	0.5950	0.0162	0.2768	0.0297	0.3182	64.71	53.48
2016	0.0446	0.5973	0.0176	0.2835	0.0270	0.3138	60.54	52.54
2017	0.0429	0.6042	0.0164	0.2896	0.0265	0.3146	61.77	52.07
2018	0.0416	0.6048	0.0161	0.2816	0.0255	0.3232	61.30	53.44
2019	0.0270	0.5765	0.0105	0.2648	0.0165	0.3117	61.11	54.07

**Table 8:** Proportion of differences in contribution in the intra-east, middle, and west regions (%).

Year	Population			Geographical Area		
	East	Middle	West	East	Middle	West
2012	48.54	33.05	18.42	3.17	0.64	96.19
2013	49.22	36.67	14.12	3.37	0.73	95.89
2014	50.90	31.84	17.26	3.24	1.23	95.53
2015	51.58	27.98	20.44	3.15	1.49	95.36
2016	51.52	27.17	21.31	3.10	1.43	95.47
2017	52.21	23.04	24.75	3.03	1.60	95.37
2018	51.96	25.49	22.55	3.11	1.54	95.36
2019	53.50	23.10	23.40	3.11	1.88	95.02

**Table 9:** Theil indices of Chinese GPs in the east, middle, and west regions.

Year	Population			Geographical Area		
	East	Middle	West	East	Middle	West
2012	0.0282	0.0192	0.0107	0.0094	0.0019	0.2856
2013	0.0251	0.0187	0.0072	0.0092	0.0020	0.2614
2014	0.0227	0.0142	0.0077	0.0090	0.0034	0.2651
2015	0.0212	0.0115	0.0084	0.0089	0.0042	0.2690
2016	0.0220	0.0116	0.0091	0.0089	0.0041	0.2738
2017	0.0213	0.0094	0.0101	0.0089	0.0047	0.2804
2018	0.0212	0.0104	0.0092	0.0089	0.0044	0.2733
2019	0.0176	0.0076	0.0077	0.0086	0.0052	0.2631

**Table 10:** The HRAD_i_ of GP allocation in 2012–2019 in China.

Area	2012	2013	2014	2015	2016	2017	2018	2019
Beijing	42.933	33.673	27.595	25.392	23.279	19.693	16.626	14.705
Tianjin	7.956	7.824	7.498	9.067	9.169	11.835	10.692	9.982
Hebei	1.605	2.333	2.525	2.483	2.257	2.000	1.845	2.544
Liaoning	1.932	1.550	1.405	1.233	1.288	1.594	1.872	1.908
Shanghai	55.943	47.239	46.300	44.970	43.969	38.770	32.250	31.366
Jiangsu	12.223	10.803	10.191	9.839	10.718	9.719	13.788	11.613
Zhejiang	10.065	10.564	10.265	10.341	9.737	10.874	7.610	6.771
Fujian	1.811	1.914	1.914	2.081	2.121	2.092	2.032	1.923
Shandong	3.734	3.206	3.140	3.182	3.291	3.248	3.415	3.486
Guangdong	3.824	4.275	4.413	4.191	4.637	4.752	4.733	4.627
Hainan	1.031	1.072	1.134	1.247	1.268	1.206	1.178	1.440
Eastern region	5.316	5.103	4.939	4.847	4.900	4.851	4.851	4.626
Shanxi	1.410	1.233	1.272	1.291	1.211	1.530	1.171	1.083
Jilin	0.558	0.574	0.663	0.762	0.805	1.010	0.800	1.027
Heilongjiang	0.398	0.417	0.454	0.481	0.447	0.373	0.383	0.379
Anhui	1.972	2.014	2.679	2.647	2.799	2.800	2.839	2.809
Jiangxi	1.080	0.951	0.997	1.002	0.992	1.187	1.037	1.046
Henan	2.470	2.537	2.793	3.151	3.332	3.538	3.813	3.581
Hubei	1.748	1.773	1.805	1.890	1.717	1.815	1.799	1.801
Hunan	1.055	1.211	1.314	1.456	1.399	1.250	1.285	2.060
Middle region	1.150	1.160	1.286	1.368	1.359	1.425	1.388	1.478
Inner Mongolia	0.127	0.135	0.141	0.136	0.977	0.131	0.132	0.132
Guangxi	1.125	1.111	1.050	0.991	0.126	0.994	1.031	1.169
Chongqing	1.718	1.737	1.692	1.759	1.728	1.768	2.376	2.569
Sichuan	0.834	1.212	1.117	1.082	0.973	0.882	0.853	0.960
Guizhou	0.507	0.560	0.756	0.900	0.959	1.071	1.090	0.956
Yunnan	0.726	0.727	0.590	0.564	0.562	0.516	0.513	0.599
Tibet	0.002	0.004	0.005	0.007	0.008	0.008	0.009	0.014
Shaanxi	0.767	0.628	0.741	0.521	0.605	0.654	0.745	0.671
Gansu	0.298	0.341	0.369	0.413	0.425	0.356	0.368	0.386
Qinghai	0.056	0.069	0.068	0.067	0.063	0.064	0.056	0.055
Ningxia	0.433	0.493	0.499	0.548	0.572	0.671	0.758	0.752
Xinjiang	0.100	0.107	0.110	0.112	0.110	0.100	0.094	0.086
Western region	0.272	0.303	0.298	0.293	0.287	0.278	0.288	0.301

**Table 11:** The HRAD_i_/PAD_i_ of GP allocation in 2012–2019 in China.

Area	2012	2013	2014	2015	2016	2017	2018	2019
Beijing	4.828	3.724	3.016	1.601	2.552	2.174	1.861	1.654
Tianjin	0.951	0.903	0.844	1.127	1.015	1.323	1.200	1.125
Hebei	0.588	0.855	0.923	0.637	0.826	0.732	0.676	0.932
Liaoning	0.924	0.745	0.679	1.361	0.632	0.789	0.934	0.958
Shanghai	2.746	2.297	2.253	1.218	2.173	1.929	1.610	1.572
Jiangsu	2.336	2.070	1.959	1.193	2.076	1.887	2.685	2.268
Zhejiang	2.746	2.887	2.815	0.976	2.665	2.959	2.054	1.801
Fujian	0.850	0.897	0.894	0.950	0.986	0.969	0.939	0.886
Shandong	0.859	0.738	0.722	1.189	0.754	0.745	0.785	0.803
Guangdong	0.920	1.029	1.060	0.868	1.100	1.117	1.102	1.066
Hainan	0.583	0.604	0.636	0.916	0.710	0.672	0.655	0.796
Eastern region	1.460	1.399	1.353	1.078	1.342	1.327	1.326	1.262
Shanxi	0.868	0.759	0.783	1.108	0.748	0.946	0.725	0.672
Jilin	0.550	0.569	0.659	0.833	0.817	1.037	0.831	1.077
Heilongjiang	0.666	0.702	0.768	0.867	0.774	0.651	0.676	0.676
Anhui	0.654	0.667	0.884	0.740	0.919	0.916	0.924	0.913
Jiangxi	0.567	0.500	0.525	1.081	0.523	0.626	0.547	0.553
Henan	0.616	0.636	0.702	0.878	0.840	0.895	0.965	0.908
Hubei	0.797	0.810	0.827	0.964	0.787	0.835	0.830	0.834
Hunan	0.477	0.547	0.592	0.806	0.630	0.564	0.580	0.932
Middle region	0.641	0.648	0.719	0.891	0.762	0.801	0.781	0.835
Inner Mongolia	0.828	0.885	0.926	0.894	0.696	0.866	0.874	0.878
Guangxi	0.809	0.797	0.752	1.077	0.832	0.706	0.731	0.827
Chongqing	0.680	0.686	0.667	1.020	0.677	0.691	0.926	0.999
Sichuan	0.709	1.032	0.952	0.745	0.828	0.751	0.727	0.819
Guizhou	0.364	0.402	0.544	0.669	0.689	0.769	0.784	0.686
Yunnan	0.846	0.847	0.688	1.231	0.655	0.601	0.598	0.698
Tibet	0.136	0.200	0.271	0.501	0.403	0.403	0.463	0.703
Shaanxi	0.597	0.489	0.579	1.030	0.474	0.513	0.583	0.526
Gansu	0.661	0.760	0.826	0.801	0.954	0.800	0.829	0.871
Qinghai	0.990	1.221	1.193	0.830	1.105	1.130	0.986	0.958
Ningxia	0.493	0.558	0.562	0.878	0.639	0.746	0.841	0.830
Xinjiang	1.058	1.118	1.142	0.927	1.107	0.996	0.926	0.834
Western region	0.714	0.798	0.784	0.911	0.751	0.728	0.752	0.787

## Data Availability

The data used for this manuscript were from the China Statistical Yearbook and the China Health and Family Planning Yearbook.

## Abbreviations

GP: general practitioner; HRA: human resources allocation; AAGR: average annual growth rate.
